# Ethylene Measurements from Sweet Fruits Flowers Using Photoacoustic Spectroscopy

**DOI:** 10.3390/molecules24061144

**Published:** 2019-03-22

**Authors:** Cristina Popa

**Affiliations:** National Institute for Laser, Plasma and Radiation Physics, Laser Department, 409 Atomistilor St., P.O. Box MG-36, 077125 Magurele, Romania; cristina.achim@inflpr.ro

**Keywords:** ethylene plant hormone, nitrogen, synthetic air, flowers respiration measurement

## Abstract

Ethylene is a classical plant hormone and has appeared as a strong molecule managing many physiological and morphological reactions during the life of a plant. With laser-based photoacoustic spectroscopy, ethylene can be identified with high sensitivity, at a high rate and with very good selectivity. This research presents the dynamics of trace gases molecules for ethylene released by cherry flowers, apple flowers and strawberry flowers. The responses of distinctive organs to ethylene may fluctuate, depending on tissue sensitivity and the phase of plant development. From the determinations of this study, the ethylene molecules at the flowers in the nitrogen flow were established in lower concentrations when the value is correlated to the ethylene molecules at the flowers in synthetic air flow.

## 1. Introduction

Laser spectroscopy-based trace gas detection has been widely applied to biology, one application of which is for ethylene measurement of animal and plant emissions [1,2,3].

The plant hormone, ethylene has emerged as a potent molecule to regulate numerous physiological and morphological responses in plants by interacting with other signaling molecules and is naturally produced by all tissues and diffused into the plant [4,5,6,7,8,9]. Ethylene plays an important regulatory role in plant responses to mineral nutrient’s availability, such as nitrogen, phosphorous, potassium, calcium, magnesium, manganese, copper, and zinc, and controls plant responses under both optimal and stressful conditions [7,10,11,12,13,14,15,16]. All tissue types and probably all cells of plants produce and liberate ethylene [1,2]. The ethylene biosynthesis and plant responses vary with the availability of mineral nutrients [4,16].

In the middle of the plant, tissues depend on the activities of known enzymes, the rate of outward dispersion and the rate of metabolism. The key to the ethylene biosynthesis is the regulation of these known enzymes [2,17,18]. Oxygen requirement in the final step involves the action of the ethylene forming enzyme (EFE) [19] and ethylene biosynthesis could be impelled by endogenous/exogenous ethylene. The activity of ethylene is not exclusively managed by endogenous ethylene concentrations in tissues, but also by the tissue sensitivity. It is generally assumed that molecules involved in ethylene in the transduction of the signal probably control how much ethylene is required to stimulate a physiological response [17,18,19,20,21,22,23,24,25,26,27,28].

Because nitrogen is a valuable nutrient required for plant development, plants are periodically exposed to nitrogen-stressed conditions, excess nitrogen due to an application of nitrogen fertilizers or insufficiency. While low nitrogen limits the growth of crop plants, the loss of excess nitrogen fertilizers contributes to environmental pollution [10,29,30,31,32]. The availability of nitrogen [4] is of agricultural concern because plant metabolism is differently affected by excess, optimal and deficient levels [10,33,34]. In maintaining the physiological status of plants under these conditions, the role of ethylene in responding to nitrogen status in plants has been identified.

This research discusses the analysis of trace gases for ethylene released by cherry flowers, apple flowers and strawberry flowers in the existence of synthetic air and nitrogen, in order to compare the responses of flowers’ tissues sensitivity.

Ethylene can be detected with laser-based photoacoustic spectroscopy, with high sensitivity and very good selectivity. In association with a flow-through system this is accepted to be combative in sensitivity and time response analogous with traditional methods such as gas chromatography, which is presently the most widely used [1]. 

Traces of ethylene reported by numerous samples absorb laser radiation inside in a photoacoustic cavity (resonant cell) and the ethylene concentration is determined from a comparison of the photoacoustic signals on various laser emission frequencies, at which ethylene has contrasting absorption strengths [35]. The laser-based instrument acknowledges detection of ethylene at sub-ppb levels with a partial pressure of 10^−10^atm and the lowest measurable concentration of 0.9 ppb [29,30].

## 2. Materials and Methods

This experimental research is dedicated to examining the effects of nitrogen and synthetic air, in the detection of ethylene at cherry blossoms, apple flowers and strawberry flowers testing photoacoustic spectroscopy with respect to the CO_2_ laser frequencies. 

For the photoacoustic detection of ethylene, I have used approximately 3 g of flowers and the analysis was carried out at room temperature. The ethylene emission was evaluated for cherry flowers, apple flowers and strawberry flowers in the presence of nitrogen and synthetic air, in order to compare the responses of flower tissues’ sensitivity.

**Figure 1:**
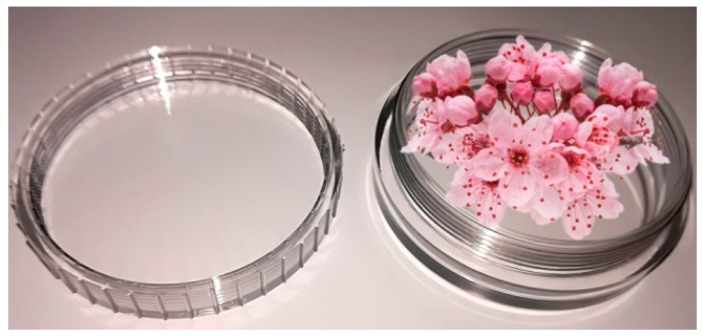
Polycarbonate capsule used for flowers.

The flowers were collected in particular colorless polycarbonate capsules used to analyze biological samples [35,36], with a specific volume of 0.83 cm^3^/g (see Figure 1).

For every polycarbonate capsule, I have added 3g of flowers, and after the collection of flowers the capsules with biological samples were introduced into a small glass cuvette (related to the resonant cell) [2].

**Figure 2:**
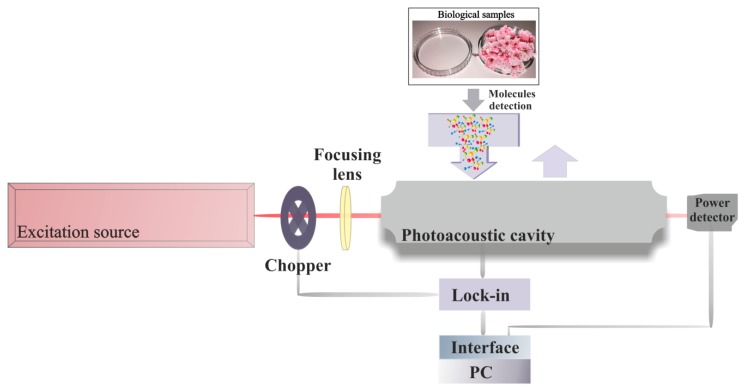
CO_2_ laser-based photoacoustic trace gas detection.

A gaseous molecule that absorbs laser radiation is excited to a higher quantum state. This absorption causes a decrease in laser light intensity, which can be directly quantified via absorption spectroscopy [1,37]. More indirect methods are observing the depopulation of the excited state via fluorescence (i.e., fluorescence spectroscopy) or by collision de-excitation. The latter gives rise to a temperature and pressure change in the gas that can be detected with a sensitive microphone (photoacoustic spectroscopy) (Figure 2) [1,38,39,40,41].

The most important optical process, as far as spectroscopic trace gas detection is concerned, is based on the extinction of radiation by molecular absorption. The absorption features and strengths specific to each molecule make it possible to identify trace gases and determine their concentrations. Absorption coefficients are typically in the order of 1 cm^−1^ (one wave number). The absorption of trace gas molecules in a gas mixture may be monitored by detecting the attenuation of the laser beam over a fixed absorption path length *L*. According to the Beer–Lambert law, the transmitted laser power in the absence of saturation is given by the following equation:

$$P\left(L\right)=P\left(0\right)\exp\left(-\alpha_{p}L\right)=P\left(0\right)\exp\left(-\alpha cL\right)$$

where *P*(0) and *P*(*L*) are the laser powers before and after the absorption cell, respectively; *α_p_* (cm^−1^) is the absorption coefficient at a given pressure of the gas at a specific laser wavelength: *α_p_* = *αc*; *α* (cm^−1^ atm^−1^) is the gas absorption coefficient (the absorption coefficient normalized to unit concentration), and *c* (atm) is the trace gas concentration [30].

The photoacoustic spectroscopy of CO_2_ laser set-up used for the evaluation of the ethylene is graphically presented in Figure 2 and completely explained in other papers [18,29,30,42]. 

Clearly, the photoacoustic detection arrangement is composed of a CO_2_ laser, a lens, a chopper, a photoacoustic cavity (resonant cell), a powermeter, a lock-in amplifier, an acquisition panel and a data-processing computer. The detection circuit is followed by a gas-handling system assembled for a suitable control of the gases molecules under study, from the gas bottle to the photoacoustic cavity. The gas handling system make sure gas purity in the resonant cell and it can be used to pump out the photoacoustic cavity, to fix up the biological sample in the cavity, monitor the total and partial pressures of gas mixtures and also, can achieve several actions without making necessary any detachments [43,44].

Practically, a CO_2_ laser-based PAS (photoacoustic spectroscopy) system utilizes a line tunable CO_2_ laser and a photoacoustic cavity, where the gas is analyzed. The cw, tunable CO_2_-laser beam is chopped, focused by a ZnSe lens, and introduced in the photoacoustic cavity. The light beam was modulated by a high quality, low vibration noise and variable speed (4–4000 Hz) mechanical chopper model DigiRad with 30 aperture blades, operated at the appropriate resonant frequency of the cell (564 Hz). The laser beam diameter is typically 6.2 mm at the point of insertion of the chopper blade and is nearly equal to the width of the chopper aperture. An approximately square waveform was produced with a modulation depth of 100% and a duty cycle of 50% so that the average power measured by the powermeter at the exit of the cell is half the cw value. By enclosing the chopper wheel in housing with a small hole (10 mm) for the laser beam to enter and exit, this reduces chopper-induced sound vibrations in air that can be transmitted to the microphone detector as noise interference. The dual-phase, digital lock-in amplifier Stanford Research Systems model SR 830 has the following characteristics: full scale sensitivity, 2 nV to 1 V; input noise, 6 nV (rms)/√Hz at 1 kHz; dynamic reserve, >100 dB; frequency range, 1 mHz to 102 kHz; time constants, 10 μs to 30 s, or up to 30.000 s. 

The resonance frequency corresponds to the resonant excitation of the first longitudinal mode of the cell (depends on its length). The acoustic resonator is characterized by the quality factor Q, which is defined as the ratio of the resonance frequency to the frequency bandwidth between half power points. The amplitude of the microphone signal is 1/View the MathML source of the maximum amplitude at these points, because the energy of the standing wave is proportional to the square of the induced pressure. For our photoacoustic cell, the profile width at half intensity was 35 Hz, yielding a quality factor Q = 16.1 at a resonance frequency.

To improve the measurement of ethylene absorption coefficients, an adapted procedure was followed. 

For every gas filling with 0.96 ppm ethylene diluted in pure nitrogen, was examined this certified gas mixture at a total pressure of approximately 1033 mbar and a temperature of 23 °C, using the commonly accepted values: 30.4 cm^−1^ atm^−1^ at 10P(14) laser transition. After measurements at all laser lines, the cell responsivity was checked (responsivity represents other parameter used to characterize the photoacoustic cell: *R = CS_M_*), to eliminate any possibility of gas desorption during the measurement. The partial pressure of ethylene was enough to have significant photoacoustic signals for all laser lines and low enough to be far away from the saturation regime. The absorption coefficient values at each laser line were obtained using the measured photoacoustic signal and laser power (the cell responsivity and ethylene concentration were known). 

To measure ethylene gas concentrations, it is necessary to adjust the cavity cell with a known gas mixture and to demonstrate the linearity of the detector signal with the concentration of the probed gas over orders of magnitude. The linear responses of the photoacoustic cavity for low detection limits of ethylene are presented substantially also in [42,43,44,45,46,47,48].

As long as the absorption coefficients of ethylene at distinct laser wavelengths were accurately calculated early [11,12,43,44,48], the CO_2_ laser was kept tuned at the 10P (14) line (10.53 μm) where ethylene exhibit a strong absorption, equivalent to an absorption coefficient of 30.4 cm^−1^ atm^−1^ ).

To examine the flower’s tissue reaction from the biological samples, was remove the extra gas, and then was cleaned the circuit. To ensure the quality of each measurement, an intensive cycle of N_2_ washing was performed between samples. After that, we transported the gas from the sample by using a synthetic air flow and a nitrogen air flow near atmospheric pressure. The ethylene emission was examined using a temperature of 23 °C and the usually established value: 30.4 cm^−1^atm^−1^.

## 3. Results and Discussion

### 3.1. Results for Flower Tissue Respiration

This practical analysis is given to examining the ethylene evolution and some specific metabolic reactions at cherry flowers, apple flowers and strawberry flowers in the nitrogen, and synthetic air conditions at room temperature using laser-based photoacoustic spectroscopy.

Ethylene biomolecules from samples were registered in nitrogen and were compared the results with flowers in the synthetic air in order to analyze the role of nitrogen in modulating the ethylene hormone response in flowers tissue.

Ethylene emission was established by introducing 3g of flowers into the biological sample for 550 s. 

The selecting parameters were used throughout the practical analysis for the determination of the ethylene gases molecules in flowers (see Table 1).

**Figure 3:**
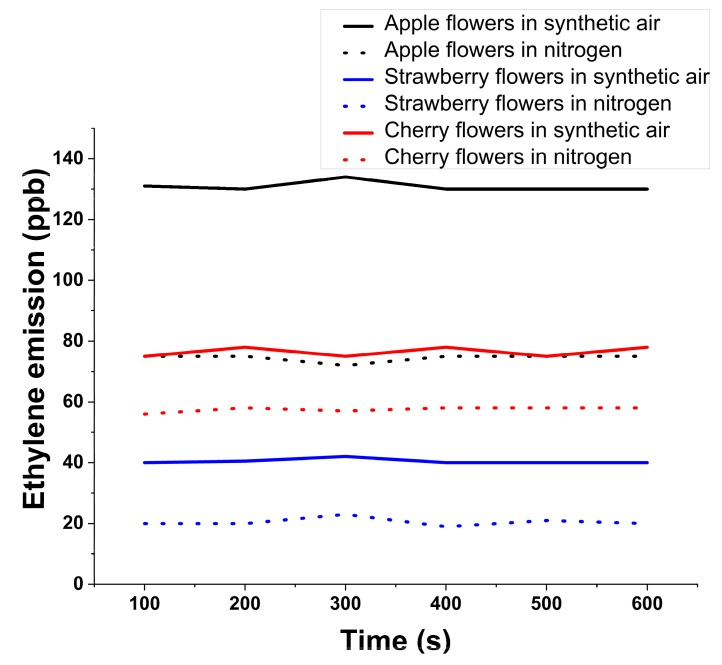
Ethylene evolution for sweet fruits flowers.

Figure 3 shows the ethylene evolution for 550 s at the sweet fruits flowers in the nitrogen flow compared to sweet fruits flowers in the synthetic air flow (used as Control).

The experiment opens with a value of around 75 ppb and after 550 s reaching a concentration of 78 ppb in synthetic air, while in nitrogen the air flow starts with a value of around 56 ppb and after 550 s the cherry flowers emit a concentration of about 59 ppb.

Ethylene respiration at strawberry flowers in synthetic air (used as Control) opens from a value of around 40 ppb and after 550 s remains at 40 ppb, while in nitrogen flow opening from a value of around 20 ppb.

For apple flowers in synthetic air, ethylene opening from a value of around 131 ppb and after 550 s presents a concentration of about 130 ppb while in nitrogen flow it opens from a value of around 75 ppb.

As can be seen, in all the cases, the value for the ethylene molecule is repressed when I have introduced the flowers in nitrogen flow; the availability of nitrogen is the main factor limiting flowers development.

Several other studies [4,49,50,51,52,53] have shown that nitrogen plays a valuable role in the development of plants, it being stated that nitrogen absence raises ethylene synthesis and tissue sensitivity [4]. Pre-harvest nitrogen absence influences the photosynthetic activity of plants, and the life of both growing and cut flowers [49]. Under conditions of damage or stress such as nitrogen deficiency, plants stimulate all the processes linked to species distribution: these, for the most part, involve the initiation of flowering in order to guarantee dissemination.

No other studies of flower senescence under conditions of nitrogen deficiency or overabundance have yet been carried out, although this would be fascinating to examine further.

Recently it was stated that nitrogen increasing of 10 mM restored ethylene levels in over-irrigated *Solanum lycopersicum* plants to the levels of well-drained plants, to an enrichment of shoot fresh weight that corresponds with reduced ethylene concentrations [50]. 

Similarly, N differentially manages proline and ethylene biostructure to mitigate salt forced synthetic restriction in mustard plants [10]. It has been also shown that extracellular ethylene increases nitrogen absorption and synthesis in *Brassica juncea* plants exposed to particular levels of nitrogen [51,52]. 

Additionally, for *Brassica *juncea**, it has been shown that plants showed insignificant photosynthesis and growth when treated with 5 mM nitrogen than 10 mM nitrogen, considering that 20 mM nitrogen was inhibitory under no-damage condition [10]. This could show that these levels were smaller and sufficient, respectively. The inhibitory response of over abundant nitrogen was linked to high ethylene presence, but under salt stress, as the requirement for nitrogen increased the ethylene and led to higher proline production and encouraged photosynthesis and growth [10]. In addition, it has been established that a high level of nitrogen constrains the lateral root growth of *Arabidopsis thaliana*, even though the number and length of lateral roots of the *etr1-3* and *ein2-1* mutants were secondarily affected than wild-type plants. The leaf long life in *Agropyroncristatum* was damaged by ethylene at distinct nitrogen levels [53]. Plants under low nitrogen conditions stimulate the development and commonly show early evolution to the reproductive phase, reaching earlier to senescence phase stage. Plants grown to high nitrogen availability have longer vegetative phase stage and slowed senescence. It is feasible to estimate that ethylene has a crucial role, since it is also known as a senescence hormone.

### 3.2. Discussion

Laser spectroscopy is very precise; a molecular transition can be measured with very high efficiency and each molecular gas has many specific lines For this argumentation, the infrared wavelength domain ranges from 9.2 to the 10.8 μm and the selection of a spectral fingerprint is based on an overall consideration of detection sensitivity, potential spectral interference and availability of laser sources; the molecule gives a unique, specific absorption pattern that can be clearly separated from other gases. Otherwise, if there is a complex mixture of gases, care has to be taken to choose a proper absorption line for the determination of the concentration, where there should be low or no interference with the absorption lines of other gases [45,54,55,56,57].

Although ethylene is not an adsorbing compound, an intensive cycle of N_2_ washing was performed between biological samples in order to have a maximum increase of 10% for the background photoacoustic signal, to ensure the quality of each measurement. It has to be underlined that the measured photoacoustic signal is due mainly to the absorption of ethylene, but some traces of CO_2_, H_2_O, ethanol, etc., influenced the measurements, with an overall contribution of<10%. The response to all absorbing species at a given laser wavelength decreased considerably when we inserted a KOH trap (with a volume >100 cm^3^), proving that amounts of CO_2_ and H_2_O vapors in the samples can significantly alter the results, thus making their removal compulsory [2].

Alongside specificity, laser spectroscopy can accomplish a very high sensitivity by growing the path length of the light through the gas. For this, mostly an absorption cavity cell is used with highly reflective mirrors, combined with advanced modulation techniques. This type of absorption spectroscopy has evolved into a wide range of methods such as cavity-enhanced spectroscopy, cavity ring down spectroscopy, wavelength modulation spectroscopy, etc.; an overview of these spectroscopic methods can be found elsewhere [58,59]. Photoacoustic spectroscopy does not need a long absorption path length, due to its intrinsically high sensitivity with high laser power [1]. Furthermore, it is an understandable and easy technique that can be used in compact and robust schemes for a gas investigation [39,40,58,59,60].

The current research describes an experimental study of the detection of ethylene released by cherry flowers, apple flowers and strawberry flowers using photoacoustic spectroscopy. These measurements are realized in different environmental condition in order to understand the dynamics of respiration of such flowers. 

Nitrogen has a stable impact on ethylene biostructure and signaling, and plants have different metabolic feedback to optimal and disturbing circumstances.

From information of the determinations of interest in the consequence of nitrogen on flowers, the nitrogen affecting the flower tissue and the ethylene plant growth regulator is repressed related to the ethylene data from the respiration of flowers in the synthetic air.

These results’ data verify and approve the anterior other investigations [4,10,50,54,55,56,57,58,59,60] that indicate that the nitrogen availability at plants and flowers affected the process respiration of ethylene with secondary effects on the prosperity and development of plants.

Nitrogen can inhibit the flowers prosperity and generation primarily correlated with the physiological, biochemical and genetic elements of the flower structure.

Our evaluations are established on the ethylene released from the respiration of flowers in two conditions: abiotic (nitrogen flow) and biotic (synthetic air flow) using laser-based photoacoustic spectroscopy and are in good agreement with those revealed in the specialized literature [48,49,50,51,52,53,54,55,56,57,58,59,60].

## 4. Conclusions

In the current research I have investigated the ethylene respiration at cherry flowers, apple flowers and strawberry flowers with nitrogen flow, and I have balanced the determinations with the ethylene respiration of flowers with synthetic air flow. 

From the determinations of this study, the ethylene molecules at flowers in the nitrogen flow were established in lower concentrations when I have correlated to the ethylene molecules at flowers in the synthetic air flow.

The results also acknowledge that the ethylene gas can be expressed as the measure of a flowers’ growth regulator; nitrogen has a substantial control on ethylene bio structure and signaling and flowers; and plants have contrasting metabolic responses to optimal and damage conditions (like abiotic condition).

As a consequence, the data from this study encourage the premise that nitrogen availabilityis a key segment in plant growth and development.

Based on a non-invasive design, safe and unchangeable in biological materials and simple to measure; the laser-based photoacoustic spectroscopy came to separate biological samples with nitrogen from biological samples with synthetic air.

The scientific literature has only recently started to examine the nature of the link between plant hormones and trace minerals signaling. 

Because the ethylene could be produced in all plant tissue and regulated by distinctive internal and external circumstances, the above work outlines current approaches in the determination of ethylene signaling route at flowers in the presence of nitrogen disturbance and synthetic air and support new data information based on photoacoustic spectroscopy. The responses of distinct organs’ structures to ethylene vary, depending on tissue responsiveness to stimuli and the phase of plant growth.

## Figures and Tables

**Table 1:** Selecting parameters for ethylene gas determination at the flowers.

Parameters	Values
Resonant cell pressure	≈1033 mb
The total amount of flowers used for determinations	≈3g
CO_2_ laser line for gas determination	10P(14); λ = 10.53 μm; α = 30.4 cm^−1^ atm^−1^
Synthetic air outflow	Linde gas: 20% oxygen, 80% nitrogen (impurities: hydrocarbons max. 0.1ppmV, nitrogen oxides max. 0.1ppmV)
Nitrogen outflow	Linde gas 6.0, purity 99.9999%
Working temperature	≈23–25 °C
Glass cell volume	150 cm^3^
Resonant cell volume	1000cm^3^
The responsivity of the resonant cell	375 cmV/W
Flowers sample time determinations	≈550 s
